# Epigenetic Modifications Related to Potato Skin Russeting

**DOI:** 10.3390/plants12102057

**Published:** 2023-05-22

**Authors:** Pawan Kumar, Yulia Kaplan, Jeffrey B. Endelman, Idit Ginzberg

**Affiliations:** 1 Institute of Plant Sciences, Agricultural Research Organization, Volcani Institute, 68 HaMacabim Road, P.O. Box 15159, Rishon LeZion 7505101, Israel; pwn.verma2008@gmail.com (P.K.); yuliakap@volcani.agri.gov.il (Y.K.); 2 Department of Horticulture, University of Wisconsin-Madison, Madison, WI 53706, USA; endelman@wisc.edu

**Keywords:** cork, DNA methylation, epigenetic regulation, periderm, phellem, phellogen, russeting, *Solanum tuberosum*, tuber skin

## Abstract

Potato tuber skin is a protective corky tissue consisting of suberized phellem cells. Smooth-skinned varieties are characterized by a clean, shiny appearance compared to the darker hue of russeted potatoes. The rough skin of russeted cultivars is a desired, genetically inherited characteristic; however, unwanted russeting of smooth-skinned cultivars often occurs under suboptimal growth conditions. The involvement of epigenetic modifiers in regulating the smooth skin russeting disorder was tested. We used smooth-skin commercial cultivars with and without the russeting disorder and three lines from a breeding population segregating for russeting. Anatomically, the russet skin showed similar characteristics, whether the cause was environmentally triggered or genetically determined. The old outer layers of the corky phellem remain attached to the newly formed phellem layers instead of being sloughed off. Global DNA methylation analysis indicated a significant reduction in the percentage of 5-methylcytosine in mature vs. immature skin and russet vs. smooth skin. This was true for both the smooth-skin commercial cultivars and the russeted lines. The expression level of selected DNA methyltransferases was reduced in accordance. DNA demethylase expression did not change between the skin types and age. Hence, the reduced DNA methylation in mature and russet skin is more likely to be achieved through passive DNA demethylation and loss of methyltransferase activity.

## 1. Introduction

Potato tubers are covered with a protective corky tissue called the periderm. The periderm comprises three cell types: phellem, phellogen, and phelloderm [1,2]. The phellem forms a series of layers at the outermost level of the periderm and is derived from the meristematic phellogen layer (or cork cambium) below it. As phellem cells develop, they become suberized and die, forming a protective corky layer termed ‘skin’. Suberin biosynthesis in the phellem has been studied extensively [3,4]. 

During tuber expansion, phellogen cell division continuously adds new skin layers, whereas superficial cork cells are sloughed off, rendering the skin smooth and shiny. Some physiological disorders of the potato tuber are related to abnormal development of the skin, including russeting of smooth-skinned potatoes. The russeted skin appears rough to the touch, with a darker tint compared to the characteristic shiny look of the smooth skin, and is often rejected by consumers. The rough skin of russeted varieties (e.g., the well-known US variety ‘Russet Burbank’) is a desired, genetically determined trait [5]. 

Impaired skin development leading to russeting is often associated with suboptimal growth conditions (i.e., environmentally triggered russeting) [6]. The phenomenon is not caused by pathogens [7], and the direct causes in the field are not clear. Nevertheless, calcium fertilization mitigates russeting occurrence and severity [7]. In contrast, increased potassium concentration in the soil solution competes with the binding of magnesium and calcium to the tuber skin and is associated with low skin quality [8]. Moreover, the disorder is enhanced in sandy soils (R. Erel, Gilat Center, Israel, unpublished data). 

Tubers with russet skin have a thicker layer of phellem than smooth-skinned potatoes [6,7,9,10]. It can result from increased activity of phellem formation—for example, due to high soil temperature [6,11]. Alternatively, thicker phellem can result from strong adherence of “old” phellem to the new layers that form below, so that they are not sloughed off during tuber development; this may be due to increased suberization [10] or increased levels of pectin and hemicellulose [9] in phellem cell walls. Nevertheless, new phellem layers are formed below the old layers, and the latter are cracked, resulting in netted or russeted skin. Whether environmentally triggered and genetically determined russeting share the same genes and biosynthesis pathways is unclear. The roughness of ‘Russet Burbank’ skin depends on growth temperatures [10] and the quantity and source of potassium fertilizer [12], suggesting that common genes might be involved.

Epigenetic factors such as DNA methylation and histone modifications were shown to play a role in phellem differentiation of cork oak, and differed between high and low phellem/cork quality [13,14,15]. Increased expression of DNA methyltransferases (DNMTs) was positively correlated with low cork quality, suggesting that gene silencing leads to a high potential for defects [15]. The russet skin disorder that develops on smooth-skin potato cultivars may be viewed as a cork of low quality [7]. 

Potato exhibits strong interaction of genotype and environment. Epigenetic regulation governs plant response to extreme growth conditions and allows phenotypic plasticity by regulating gene expression levels [16,17]. The potato periderm is a protective tissue enriched with stress-related factors [18] and responds to stress conditions. For example, temperature stress enhanced the formation and accumulation of skin layers to create a thick protective cover [6]—this demonstrates the strong impact of environmental cues on cork development and the potential for epigenetic modulation of its quality. Agronomical practices may also induce chromatin modifications that affect skin quality [19,20]. 

Epigenetic mechanisms change the phenotypic traits in the plant without changing the genetic sequences. Examples are DNA methylation, histone modifications, and histone variants. DNA methylation is based on a dynamic addition or removal of a methyl group. In plants, it occurs at the fifth position of the cytosine ring in three contexts: CG, CHG, and CHH (H = C, T, or A), and is mediated by the RNA-directed DNA methylation (RdDM) pathway, involving small interfering RNAs (siRNAs) and scaffold RNAs in addition to protein complexes [21]. Four DNMT families differentially regulate cytosine methylation. METHYLTRANSFERASE1 (MET1) recognizes hemimethylated CG following DNA replication and methylates the naked cytosine in the daughter strand [21,22]. Chromomethylases (CMTs) bind histone H3 lysine 9 (H3K9me2) heterochromatin to methylate non-CG contexts [23]. In flowering plants, CMT3 methylates mostly CHG sites, whereas CMT2 methylates mostly CHH sites [24,25]. The CHH methylation state by CMT2 is maintained by DOMAIN REARRANGED METHYLTRANSFERASE 2 (DRM2) through the RdDM pathway [25,26].

Passive DNA demethylation results from an absence of DNMTs, a reduction in their activity, or a shortage of methyl donors following DNA replication [27]. In plants, active DNA demethylation is mediated by 5-methylcytosine DNA glycosylases through a DNA base-excision repair pathway [28]. There are four 5-methylcytosine DNA glycosylases in *Arabidopsis*: REPRESSOR OF SILENCING 1 (ROS1), DEMETER (DME), DEMETER-LIKE 2 (DML2), and DEMETER-LIKE 3 (DML3) [28]. 

To test whether epigenetic modification is involved in potato skin maturation and russeting, we used smooth-skin potato commercial cultivars with and without the russeting disorder (environmentally triggered russeting), in addition to three lines from a breeding population segregating for russeting (i.e., genetically determined russeting)—one smooth and two russeted to different levels. An anatomical study was used to define the different russeting phenotypes, global DNA methylation (GDM) was determined for smooth and russet skin types, and the expression level of selected DNA modifier genes was monitored. 

## 2. Results

### 2.1. Description of Russet Skin

**Figure 1:**
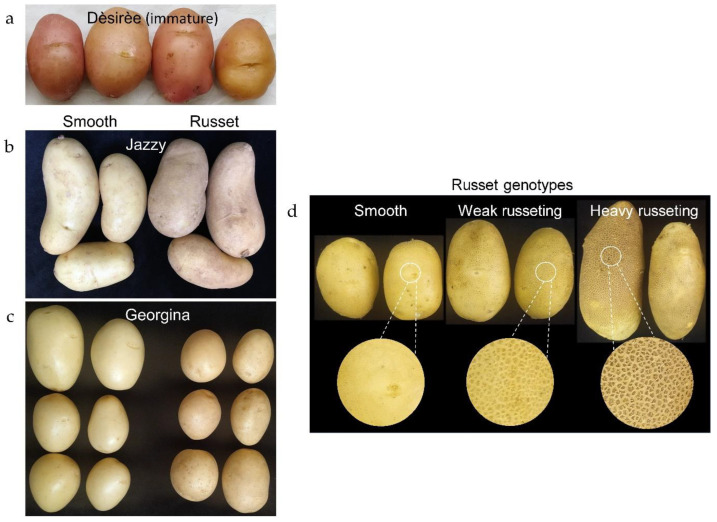
Images of potatoes used in the study. (**a**) The red cultivar Dèsirèe at immature stage. Rosanna tubers are not shown here but look similar. (**b**,**c**) Tubers of the white cultivars Jazzy and Georgina, respectively. On the left of each frame are tubers with the characteristic shiny and smooth skin; on the right are tubers with environmentally triggered russeting. (**d**) Three lines from a breeding population segregating for russeting; left to right (in pairs) are tubers with smooth-skin, weak russeting, and heavy russeting. Enlarged skin images are given below the respective tubers.

The smooth-skinned cultivars used in the study are characterized by a clean, shiny appearance (Figure 1a–c); Dèsirèe and Rosanna also exhibit a red/purplish tint due to the accumulation of anthocyanins in their periderm. Skin with environmentally triggered russeting that develops on the surface of the cultivars Jazzy and Georgina appears with a darker hue without the shiny look, as well as fine cracking (Figure 1b,c). The russet on the surface of the 43_wR and 25_hR lines is coarser, rough to the touch, and with netting-like morphology (Figure 1d); the latter exhibits a darker tint than the former. 

### 2.2. Anatomical Study of Russeted Skin

**Figure 2:**
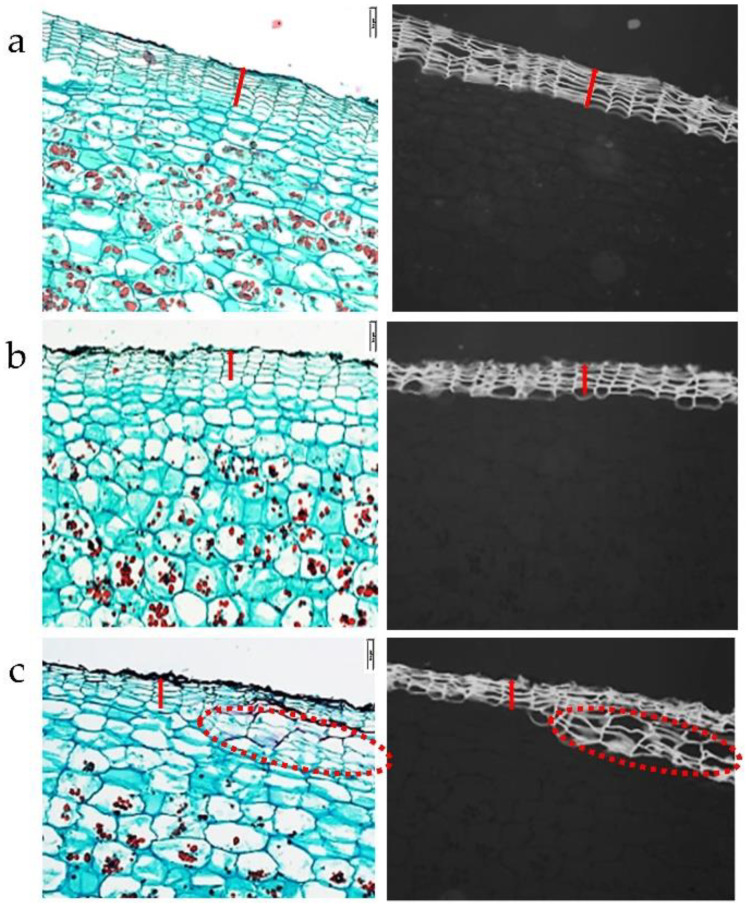
Environmentally triggered russeting. Anatomy of smooth and russet skin of potato cultivars. (**a**) Smooth skin of cv. Rosanna, (**b**) smooth skin of cv. Jazzy, (**c**) russet skin of cv. Jazzy with newly formed phellem layers (encircled) below it. The left and right panels are images from light and UV microscopes, respectively. The red bar marks the phellem layers (the skin). Scale bar = 100 µm.

An anatomical study was conducted to distinguish between the morphologies of the two skin types, smooth vs. russet. Sections through the surface of Rosanna tuber demonstrate typical morphology of the skin made of phellem cells. The phellem are rectangular flattened cells arranged in columns of ca. ten layers, and their suberized cell walls are auto-fluorescing under UV light (Figure 2a).

Smooth skin of cv. Jazzy shows similar morphology of skin organization, albeit fewer cell layers (ca. five layers; Figure 2b). The russet skin of Jazzy seems more condensed than the smooth skin. New phellem cell layers are formed underneath the russet layers, although the skin was already cured and adhering firmly to the tuber flesh (Figure 2c). The old phellem cells remain attached to the newly formed skin layers. During normal skin development, these cells are sloughed off, rendering the skin smooth and shiny.

**Figure 3:**
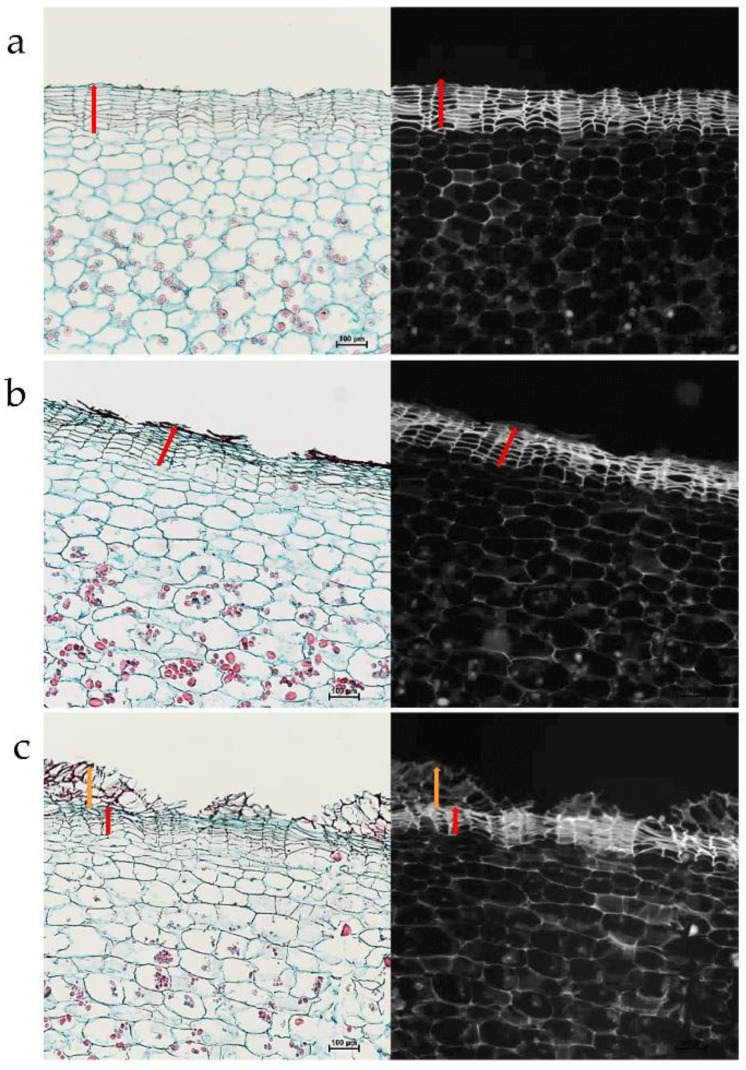
Genetically determined russeting. Anatomy of smooth and russet skin of potato breeding lines. Lines with smooth (**a**), weak russeted (**b**), and heavy russeted skin (**c**). The left and right panels are images from light and UV microscopes, respectively. The red bar marks the phellem layers. The orange bars mark the russet layers. Scale bar = 100 µm.

The skin of the smooth line, 16_S, exhibits the characteristic phellem cell columns that are nicely arranged and with numerous cell layers (Figure 3a). The russet skin of lines 43_wR and 25_hR has patches of dead phellem that adhered to the newer phellem layers underneath them—in 43_wR with weak russeting, this phenomenon is finer compared to 25_hR with heavy russeting (Figure 3b,c, respectively). Note the autofluorescence signal of the russet skin fragments under UV light is weaker than that of the native phellem. 

### 2.3. Global DNA Methylation of Russeted Skin

**Figure 4:**
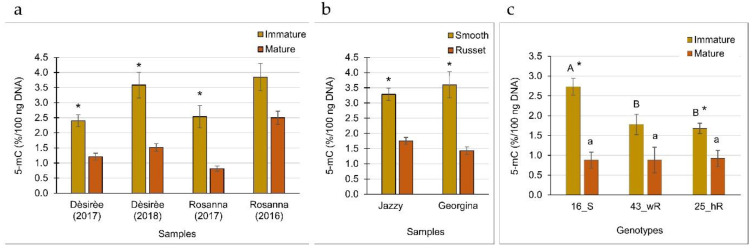
Global DNA methylation level in tuber skin, (**a**) at two developmental stages, (**b**) following environmentally triggered russeting, and (**c**) in the skin of russet and smooth breeding lines. (**a**) Samples were collected at immature and mature developmental stages from tubers of Dèsirèe and Rosanna cultivars for two successive years. (**b**) Smooth and russet regions of the skin were collected from tubers of two potato cultivars, Jazzy and Georgina. (**c**) The skin was collected from breeding lines with smooth skin (16_S), weak russeting (43_wR), and heavy russeting (25_hR). Each bar represents the averages of three biological replicates with SE. Asterisks indicate statistical significance between two means of the same sample. Capital letters compare the immature skin of the breeding lines, and small letters are for mature skin. Statistical analysis was done by Tukey-Kramer HSD (*p* < 0.05).

To examine whether russeting is associated with epigenetic regulation of gene expression, GDM was determined in DNA extracted from russet and smooth skin. First, a comparison was made between the immature and mature skin of smooth cultivars Dèsirèe and Rosanna to monitor the status of the GDM at two developmental stages of the skin. This was done for two years for each cultivar. Results showed a significant reduction in GDM status in mature skin compared to immature skin (Figure 4a). 

Comparison of the smooth and russet skin of Jazzy and Georgina showed a significant reduction in GDM levels in the russet skin (Figure 4b). Reduced GDM levels were also monitored using the breeding lines 16_S, 43_wR, and 25_hR—each line showed significantly lower levels in the mature compared to immature developmental stage of the skin, irrespective of smooth or russet skin type (Figure 4c). Moreover, in the stage of skin immaturity, the russet skin type of 43_wR, and 25_hR had reduced GDM levels compared to the smooth skin type of 16_S (Figure 4c). Thus, the results obtained for the tested breeding lines supported the previous data obtained for the smooth skin cultivars with and without the environmentally triggered russeting—reduced GDM levels in maturing and in russeted skin. 

### 2.4. Expression of Epigenetic Modifiers in Russeted Skin

**Figure 5:**
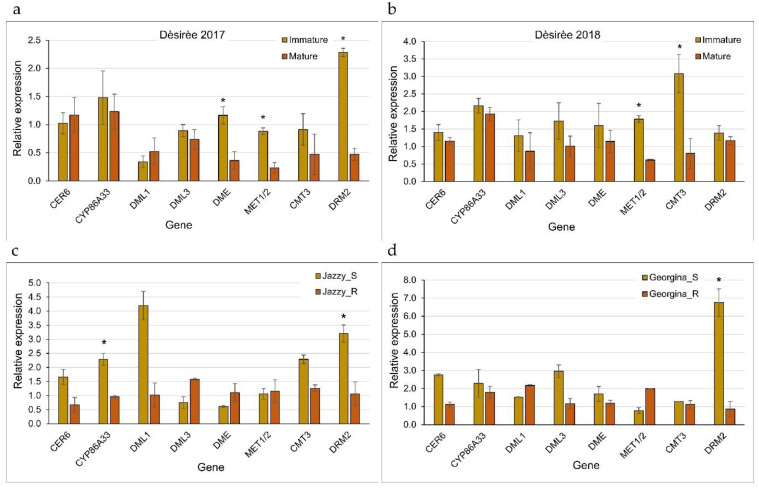
The expression level of genes coding for epigenetic modifiers that may determine skin maturation and russeting (listed in Table 1). (**a**,**b**) Immature and mature skin of the cultivar Dèsirèe collected in 2017 and 2018, respectively. (**c**,**d**) Smooth (S) and russet (R) skin of the cultivars Jazzy and Georgina, respectively. Each bar represents the averages of three biological replicates with SE. Asterisks indicate statistical significance between two means of immature vs. mature or smooth vs. russet. Statistical analysis was done by Tukey-Kramer HSD (*p* < 0.05).

A literature survey was conducted to identify genes coding for DNMT and DNA demethylases that may be responsible for the DNA epigenetic modification associated with russeting—both the environmentally triggered and genetically determined traits. Similar genes were also identified in previous work on potato phellogen transcriptome (Table S1; [11]). Gene expression of selected epigenetic modifiers was tested in the same skin samples as above. This included the DNA demethylases *DML1*, *DML3*, and *DME*, and the DNMTs *MET1*/*2*, *CMT3*, and *DRM2*. The expression of two markers for suberization, *CER6*, and *CYP86A33*, was used as a reference to phellem cell activity. A comparison of Dèsirèe’s immature and mature skin over two years indicated similar activity of the suberization markers in both developmental stages (Figure 5a,b). *Met1/2* was significantly downregulated in the mature skin in both years; *DRM2* and *DME* were strongly downregulated in 2017 and *CMT3* in 2018. When gene expression was monitored in the smooth vs. russet skin of Jazzy and Georgina, results indicated reduced expression of suberization genes in the russet skin, but the differences were not always statistically significant (Figure 5c,d). In both cultivars, *DRM2* was significantly downregulated in the russet skin.

**Figure 6:**
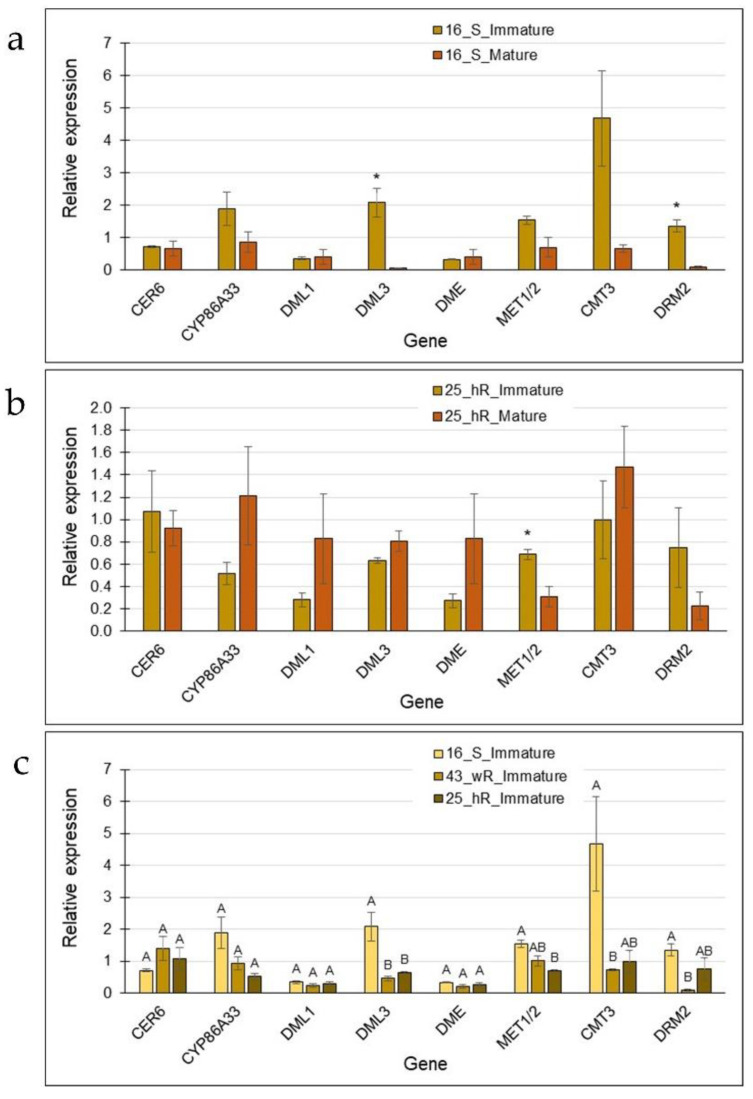
The expression level of genes coding for epigenetic modifiers that may determine skin maturation and russeting in immature and mature skin of smooth and russeted genotypes. (**a**) The smooth genotype 16_S; (**b**) the heavy russeted genotype 25_hR; and (**c**) a comparison of the immature state of the three genotypes, the smooth 16_S, the weak russeted 43_wR, and the heavy russeted 25_hR. Each bar represents the averages of three biological replicates with SE. Significant difference among means is indicated by an asterisk or different letters. Statistical analysis was done by Tukey-Kramer HSD (*p* < 0.05).

Comparisons of immature vs. mature skin for the smooth line 16_S and for the russeted line 25_hR pointed again at the association of reduced expression of *MET1*/*2* and *DRM2* in mature skin, albeit not always significantly (Figure 6a,b). *DML3* was also downregulated in the mature skin of 16_S (Figure 6a). When all three breeding lines were compared at their immature stage (when skin formation is most active), *DML3* was significantly reduced in the russeted genotypes. *CMT3* and *DRM2* were significantly reduced in 43_wR and *MET1*/*2* in 25_hR when compared to the smooth genotype 16_S (Figure 6c). Overall, it can be concluded that skin aging and russeting are similarly associated with reduced activity of DNMTs, resulting in reduced GDM levels in the respective skin types.

## 3. Discussion

Environmentally triggered russeting of smooth skin cultivars and genetically determined russeting appear as brownish regions on the surface of the tubers. Tissue color or roughness, and the degree of surface coverage, may differ between genotypes, but russet tissue morphology has a common characteristic: the old outer layers of the corky phellem remain attached to the newly formed phellem layers instead of being sloughed off. These superficial layers have a compressed structure that is less organized than the phellem layers of the smooth skin type, and they crack as the tuber expands [6,7]. The outcome is russeted skin that looks like flakes of dry skin adhered to the surface of the tuber or as netted rough skin.

During recent years, we applied diverse experimental approaches to cope with potato skin disorders, including various protocols for haulm desiccation [29] and mineral fertilization [7,8,30]. These were complemented with proteomic and transcriptomic studies of skin development [6,18,31] and the identification of skin/suberin-related genes [32]. Although the treatments we have deployed slightly improved skin appearance, none “cured” the costly problem of the environmentally triggered russeting in smooth skin cultivars. This motivated us to look for an epigenetic mechanism that may indicate tuned regulated expression of known genes during russet development and of unknown regulating sequences that could not be detected by the omics approaches we deployed previously. 

GDM analysis of potato skin indicated a significant reduction in the percentage of 5-methylcytosine in maturing skin compared to immature skin, and in russet compared to smooth skin. This was true for smooth skin commercial cultivars Dèsirèe, Rosanna, Jazzy, and Georgina and the breeding lines segregating for russeting. In accordance, the gene expression level of selected DNMTs was reduced in mature vs. immature skin and in russet vs. smooth skin in all the potatoes tested. In most cases, *DRM2* was dramatically downregulated, albeit not always significantly. *MET1*/*2* was mainly downregulated in mature vs. immature smooth skin and in genetically determined heavy russeting but not in environmentally triggered russeting. The *CMT3* expression profile was not conclusive among the tested samples. Of the DNA demethylases, *DML3* was downregulated in maturing smooth skin of 16_S and the russeted lines compared to the smooth line. 

DNA demethylation at a subset of regions relies on active DNA demethylation initiated by DNA glycosylases *ROS1*, *DML2*, and *DML3* but can also occur passively [33]. In potato skin, reduced DNA methylation level in aging or russeted skin is more likely to be achieved through passive DNA demethylation and loss of DNMT activity.

Overall it is concluded that skin aging and russeting involve reduced DNA methylation associated with passive demethylation and reduced activity of DNMTs.

Tuber skin is a model system for plant corky tissues and may be compared to the cork of trees. Epigenetic factors such as DNA methylation and histone modification were shown to play a role in cork differentiation of cork oak (*Quercus suber*) and with altered patterns between cork of high and low quality [13,14,15]. Orthologs of genes involved in epigenetic modifications of oak phellogen and cork quality were found in the transcriptome of potato skin phellogen [11]. In general, the russet skin of smooth potato cultivars can also be viewed as a cork of low quality [7].

In potato skin, DNA methylation level and *DRM2* expression were positively associated—high *DRM2* gene expression and high DNA methylation level—while the contribution of other DNMT activities seems less consistent between samples. These high activities were associated with smooth skin (corky skin of high quality). In accordance, in the cork of *Q. suber, DRM2* was the most active methyltransferase compared to *MET1*, *MET2*, and *CMT3* [15]. However, contrary to what was shown for the potato skin, *Q. suber* cork of high quality was associated with lower levels of DNMTs gene expression. Yet, the trend of GDM was similar in potato and *Q. suber*—high in cork of high quality. Thus, in *Q. suber* there was a negative correlation between the relative levels of expression of DNMTs and GDM [15]. Nevertheless, it was suggested that gene silencing is higher in cork of low quality, leading to a higher potential of defects [15].

Conversely, in potato, the level of GDM and related gene expression were lower in maturing and russeted skin (corky skin of low quality). Therefore, it can be speculated that skin maturation requires fewer cellular specifications, hence less gene silencing by methylation, compared to the early stages of skin formation. Moreover, reduced gene regulation by methylation leads to “abnormal” development, hence to russeting. 

DNA methylation provides stringent regulation or fine-tuning of gene expression in controlling phenotypic variation in response to environmental factors or at critical developmental stages [17,27]. In potato, increased DNA methylation was reported for photoperiod-sensitive tuberization [34] and in response to heat [35] and salt stress [36]. Accordingly, we hypothesize that reduced DNA methylation in aging or russeted skin reflects weak control of skin developmental processes and, thus, the formation of russeted skin instead of smooth. This is further demonstrated when we examine skin/periderm developmental stages. 

The transcriptome of potato phellogen included DNA markers for epigenetic regulation [11]. The highest expression (given by FPKM) was related to genes involved in chromatin assembly/remodeling, as expected for cambial tissue that is extensively dividing. Components of RdDM pathway were also expressed, however, to a lesser extent. This included *DRM2*, *AGO4*, *DDM1*, *DRD1*, and *RDR2* [11,25,27]. The expression of *DML1*/*ROS1* with demethylation activity was very low, as well as histone demethylation activity. Transcripts for *MET1*, *MET2*, and *CMT3* that maintain the methylation sites were not detected at that early stage of development. Overall, it is suggested that in the phellogen, initiation of methylation activity allows the specific programming of developmental pathways towards the formation of suberized phellem (skin; external layers of the periderm) and the parenchyma-like phelloderm (internal periderm layers). Thus, in smooth skin cultivars, high DNA methylation directs the formation of the skin of high quality. Reduction in DNA methylation suggests a change of program or looseness of controlled processes that leads to russeting.

Data also imply that skin russeting may share a similar DNA methylation state as the skin maturation process. Whether the same set of structural and regulatory genes are epigenetically regulated requires further clarification.

## 4. Materials and Methods

### 4.1. Plant Material

The study included four commercial cultivars of potato (*Solanum tuberosum* L.)—Dèsirèe, Rosanna, Jazzy, and Georgina—and three UW-Madison breeding lines from North American russet germplasm, which were selected from two populations segregating for russeting: W17071 = AW07791-2rus x AW07791-2rus; and W17069 = AW06108-rus x AW07791-2rus. The three breeding lines were as follows: W17069–16 (abbreviated 16_S) had smooth skin; W17071-43 (abbreviated 43_wR) had weak russeting; and W17071-25 (abbreviated 25_hR) had heavy russeting (Figure 1). Dèsirèe and Rosanna are red-skin cultivars and were used in this work interchangeably. 

Tubers of Jazzy and Georgina were sampled from commercial fields in the northern part of the Negev region of Israel at harvest time in 2019–2020. These were sorted into two groups—tubers with the cultivar characteristic smooth skin and tubers with an environmentally triggered russeting. Plants of Dèsirèe, Rosanna, and the 16_S, 43_wR, and 25_hR genotypes were grown in 20-L containers in a greenhouse in Volcani Institute, in 2016-2018 and 2022, respectively, under natural winter conditions (November–January, average temperature range of 10–18 °C). Tubers were collected at two developmental stages—at 7–8 weeks post sprout emergence when the skin is immature and can be easily separated by the hand from the tuber flesh, and at 10–12 weeks post sprout emergence during skin maturation and skin-set. For each cultivar and the breeding lines, three individual plants were harvested at each time point. Tubers of the same plant were pooled for sampling. 

At sampling, tissue blocks were taken from the tuber surface for anatomical study of the skin, and skin tissue was collected for the molecular analyses described below. The latter was frozen in liquid nitrogen and stored at −80 °C until use. 

### 4.2. Anatomical Studies

Tissue blocks from the surface of the tubers were fixed in FAA (50% ethanol, 5% acetic acid, and 3.7% formaldehyde, *v*/*v*, in water), dehydrated in an ethanol/xylene series, and embedded in paraplast (Surgipath Paraplast Plus, Leica Biosystems Richmond Inc., Richmond, IL, USA), according to standard methods [37]. Tissue sections (16 µm) were stained with Safranin-O/Fast green (Sigma-Aldrich, Jerusalem, Israel) [38] and were observed under the microscope (Leica DMLB, Germany) through a CCD camera (Leica DC2000) by using the Leica IM1000 program. Visible light was used to examine skin tissue morphology, and UV light to view the autofluorescence of the phellem-suberized cell walls. 

### 4.3. Total DNA Extraction and Global DNA Methylation (GDM) Assay

Total DNA was extracted from skin tissue using the DNeasy^®^ Plant Pro Kit (QIAGEN GmbH, Germany). Purified DNA was quantified in the NanoDropTM 2000/2000c (Thermo Scientific, Waltham, MA, USA). Determination of GDM was performed using the MethylFlash™ Global DNA Methylation (5-mC) ELISA kit (Colorimetric) (Epigentek, USA catalog no. # P-1030). Tested genomic DNA was used at 100 ng per reaction according to the kit protocol. A standard curve was prepared with a solution of methylated DNA containing 5% of 5-methylcytosine at 5 ng/μL and 50 μg/mL. The negative control was a solution of unmethylated DNA. Both solutions were provided in the kit. According to the assay protocol, a standard curve was prepared at 0, 0.2%, 1%, and 5% of 5-methylcytosine using the standard solutions. Absorbance at 450 nm was recorded using the Synergy H1 Hybrid Reader Photometer (BioTek, Agilent Technologies Inc., Santa Clara, CA, USA). Analyses were performed with three biological replicates, each with two technical replicates. Data were analyzed for significance by Tukey’s HSD test (*p* < 0.05) using the JMP16 software (http://www.jmp.com) 

### 4.4. Total RNA Extraction, cDNA Synthesis, and Quantitative Reverse Transcriptase PCR (qRT-PCR)

Total RNA was extracted from skin samples according to Ginzberg et al. [6]. cDNA synthesis was done using QuantiTect Reverse Transcription Kit (QIAGEN GmbH, Germany). LightCycler^®^ 480 SYBR Green I Master (Roche Diagnostics GmbH, Mannheim, Germany) was used for qRT-PCR according to the manufacturer’s protocol with specific primers (Table 1). Gene expression data was quantified using a standard curve—a mixture of the tested cDNA samples at equal amounts was diluted 1:30, 1:240, and 1:1920 and used in each qRT-PCR with the specific primers. Reactions with the individual tested cDNAs and the mixed samples for the standard curve were run in a Rotor-Gene 6000 Real-Time PCR machine (Corbett Research Ltd., Sydney, NSW, Australia), and the build-in software calculated relative gene expression values. Each qRT-PCR was performed with three biological replicates, each with three technical replicates. Values in each sample were normalized to the expression levels of the reference gene, *α-CHAIN OF THE NASCENT POLYPEPTIDE-ASSOCIATED COMPLEX* (*α-NAC*) [6]. Data were analyzed for significance by Student’s *t*-test (*p* < 0.05) using the JMP16 software (http://www.jmp.com).

**Table 1:** Primers used in the study.

Gene Code	Gene	Potato ID	Forward Primers	Reverse Primers
*DML1*	*DEMETER-LIKE DNA GLYCOSYLASE 1*	Soltu.DM.09G004240	CCACCAAATGTTTCTCGACC	TGTTGTGGCTTCAATACCCT
*DML3*	*DEMETER-LIKE DNA GLYCOSYLASE 3*	Soltu.DM.04G017430	CTGAGCACACAGTTTTGAGG	ATTGCTCTCAGCCTGTAACC
*DME*	*DNA GLYCOSYLASE DEMETER*	Soltu.DM.11G005260	ATTCAAAGGCCTAACCACAG	CTGGCTTTCCTTTTGTCCTA
*MET1*	*DNA (CYTOSINE-5)-METHYLTRANSFERASE 1*	Soltu.DM.11G013230	TAAGGTGGGGATGTGCTTTC	GTGCATATGCCAAAGGAGGT
*CMT3*	*CHROMOMETHYLASE 3*	Soltu.DM.12G000130	ACTTGCCTGGAGTTCGTGTT	GTAGTTCCTCAAAACCGTTT
*DRM2*	*DOMAINS REARRANGED*	Soltu.DM.02G006560	GGATCAGGAAAGCTGTGGAG	GGTTCTTTGGAAACCCCAAT
*NAC*	*NASCENT POLYPEPTIDE-ASSOCIATED COMPLEX*	Soltu.DM.09G002650	ATATAGAGCTGGTGATGACT	TCCATGATAGCAGAGACTA

## Data Availability

The data presented in this study is contained within the article and the Supplementary Material Table S1.

## Supplementary Materials

The following supporting information can be downloaded at: https://www.mdpi.com/article/10.3390/plants12102057/s1. Table S1. Epigenetic markers in the transcriptome of potato phellogen.
