# Supplementary material for: Epigenetic Modifications Related to Potato Skin Russeting
Source: Plants (Basel). 2023 May 22;12(10):2057. doi: 10.3390/plants12102057 (PMC10222780; doi:10.3390/plants12102057)
Supplement: Supplementary file 1 [file plants-12-02057-s001.zip › plants-2312703-supplementary.pdf]

Table S1. Epigenetic markers in the transcriptome of potato phellogen †

Gene code	Gene name	Comment	FPKM	Sotub ID	TAIR ID
**Chromatin assembly/disassembly/remodeling**					
*SWC4**	SWR1-COMPLEX 4			Sotub01g023480.1.1	At2g47210
HMGB3	HIGH MIBILITY GROUP B3		2117.77	Sotub02g026070.1.1	At1g20696.3
HMGB3	HIGH MIBILITY GROUP B3		1774.75	Sotub03g008810.1.1	At1g20696.2
CHR4	CHROMATIN REMODELING 19		818.05	Sotub02g014700.1.1	At5g44800.1
CHR19	CHROMATIN REMODELING 11		284.21	Sotub02g005500.1.1	At2g02090.1
CHR11	CHROMATIN REMODELING 11		6.64	Sotub01g021440.1.1	At3g06400.2
EEN	CHROMATIN REMODELING COMPLEX		2.57	Sotub06g006620.1.1	At4g38495.1
CHC1	CHROMATIN REMODELING COMPLEX ASSOCIATED WITH SWI/SNF		99.45	Sotub02g014170.1.1	At5g14170.1
CHC1	CHROMATIN REMODELING COMPLEX ASSOCIATED WITH SWI/SNF		1.37	Sotub03g023850.1.1	At5g14170.1
**DNA methylation pathways**					
*DRM2**	DOMAINS REARRANGED METHYLTRANSFERASE		35.17	Sotub02g010360.1.1	At5g14620
AGO4	ARGONAUTE 4		408.96	Sotub01g009760.1.1	At2g27040.2
DDM1	NUCLEOSOME REMODELER		0.62	Sotub02g010390.1.1	At5g66750.1
DRD1	RNA-DIRECTED DNA METHYLATION 1		0.24	Sotub01g047420.1.1	At2g16390.1
RDR2	RNA-DEPENDENT RNA POL 2		466.40	Sotub03g027500.1.1	At4g11130.1
ELF6	EARLY FLOWERING 6		763.84	Sotub04g018130.1.1	At5g04240.1
**DNA demethylation**					
DML1, ROS1	DEMETER LIKE1		0.48	Sotub03g034510.1.1	At2g36490.1
**Histone (de)methylation**					
*ATX4**	ARABIDOPSIS TRITHORAX	H3K4me3	3.53	Sotub01g033330.1.1	At4g27910
*ATXR4**	ATX-Related	H3K4me3	0.68	Sotub03g011170.1.1	At5g06620
*SUVH4, KYP**	SU(VAR)3-9 HOMOLOG, KRYPTONITE	H3K9me1	169.83	Sotub02g037730.1.1	At5g13960
*SUVH6**			1.12	Sotub03g016470.1.1	At2g22740
AL7	ALFIN-LIKE 7 di- or trimethylated histone H3 (H3K4me3/2)	H3K4me3/2	1265.27	Sotub01g039670.1.1	At1g14510.1
IBM1	INCREASE IN BONSAI METHYLATION 1	H3mK9 demethylation	228.24	Sotub04g016780.1.1	At3g07610.1
JMJ20	JUMONJI DOMAIN-CONTAINING PROTEIN 20	H4-R3 demethylation	18.47	Sotub11g022090.1.1	At5g63080.1
JMJ16	JUMONJI DOMAIN-CONTAINING PROTEIN 16	H3K4 and H3K9 demethylation	8.29	Sotub04g009950.1.1	At1g08620.2
JMJ26	JUMONJI DOMAIN-CONTAINING PROTEIN 26	H3K9 demethylation	2.61	Sotub02g024130.1.1	At1g11950.1
JMJ24	JUMONJI DOMAIN-CONTAINING PROTEIN 24	H3K9 demethylation	1.65	Sotub02g021650.1.1	At1g09060.3
JMJD5	JUMONJI DOMAIN-CONTAINING PROTEIN D5	Histone demethylase	0.07	Sotub01g007240.1.1	At3g20810.1
**Histone deacetylase**					
*HDA3**	HISTONE DEACETYLASE 3		8.25	Sotub11g024650.1.1	At3g44750
HDA5	HISTONE DEACETYLASE 5		50.12	Sotub03g028680.1.1	At5g61060.1
HDA15	HISTONE DEACETYLASE 15		1.41	Sotub03g033280.1.1	At3g18520.1
SNL2	SIN3-LIKE 2		4.52	Sotub02g035490.1.1	At5g15020.2
**Histone acetyltransferase**					
HAC1	HISTONE ACETYLASE 1		1021.46	Sotub01g008490.1.1	At1g79000.2
**Known epigenetically regulated genes related to corky skin**					
H1.3	HISTONE1.3		26.96	Sotub02g027480	At2g18050
MYB1	MYB TRANSCRIPTION FACTOR	Repression of lignin biosynthesis	6.84	Sotub01g048880	At4g38620

† (Vulavala et al. 2019)

* Putative regulators of *Q. suber* cork quality (Inácio et al. 2018)
